# Body mass index and cardiovascular outcomes in patients with acute coronary syndrome by diabetes status: the obesity paradox in a Korean national cohort study

**DOI:** 10.1186/s12933-020-01170-w

**Published:** 2020-11-10

**Authors:** Se-Jun Park, Kyoung Hwa Ha, Dae Jung Kim

**Affiliations:** 1 Division of Cardiology, Department of Internal Medicine, GangNeung Asan Hospital, University of Ulsan College of Medicine, Gangneung, Korea; 2 Department of Endocrinology and Metabolism, Ajou University School of Medicine, 164 World Cup-ro, Yeongtong-gu, Suwon, 16499 Korea; 3 Ajou University School of Medicine, Cardiovascular and Metabolic Disease Etiology Research Center, Suwon, Korea; 4 Department of Cardiology, Armed Forces Capital Hospital, Seongnam, Korea

**Keywords:** Obesity, Obesity paradox, Diabetes mellitus, Acute coronary syndrome

## Abstract

**Background:**

The “obesity paradox” has not been elucidated in the long-term outcomes of acute coronary syndrome (ACS). We investigated the association between obesity and cardiovascular (CV) outcomes in ACS patients with and without diabetes.

**Methods:**

We identified 6978 patients with ACS aged 40–79 years from the Korean National Health Insurance Service-Health Screening Cohort between 2002 and 2015. Baseline body mass index (BMI) was categorized as underweight (< 18.5 kg/m^2^), normal weight (18.5–22.9 kg/m^2^), overweight (23.0–24.9 kg/m^2^), obese class I (25.0–29.9 kg/m^2^), and obese class II (≥ 30.0 kg/m^2^). The primary outcome was major adverse CV events (MACE)—CV death, myocardial infarction (MI), and stroke. The secondary outcomes were the individual components of MACE, hospitalization for heart failure (HHF), and all-cause death.

**Results:**

After adjustment for confounding variables, compared to normal-weight patients without diabetes (reference group), obese class I patients with and without diabetes had a lower risk of MACE, but only significant in patients without diabetes (with diabetes: hazard ratio [HR] 0.95, 95% confidence interval [CI] 0.78–1.14; without diabetes: HR 0.78, 95% CI 0.62–0.97). Obese class II patient with diabetes had a higher risk of MACE with no statistical significance (HR 1.14, 95% CI 0.82–1.59). Underweight patients with and without diabetes had a higher risk of MACE, but only significant in patients with diabetes (with diabetes: HR 1.79, 95% CI 1.24–2.58; without diabetes: HR 1.23, 95% CI 0.77–1.97).

**Conclusion:**

In ACS patients, obesity had a protective effect on CV outcomes, especially in patients without diabetes.

## Background

Obesity is a well-established risk factor for cardiovascular (CV) disease that increases the risk of CV mortality [1, 2]. However, several studies have reported that obese patients with high body mass index (BMI) have a better prognosis than patients with normal BMI [3–7]. However, the mechanism of this phenomenon, called “obesity paradox” is unclear.

Diabetes mellitus has been known to increase the risk of CV disease, and patients with diabetes experience worse CV outcomes than those without diabetes [8]. In a prospective cohort study, diabetes increased the risk of mortality by 140% in patients with previous myocardial infarction (MI) [9]. Diabetes was also associated with 30-day and 1-year mortality in ACS patients [6].

There has been an ongoing debate regarding the relationship between obesity and CV outcomes according to diabetes status [10]. In a large-scale population study regarding diabetes mellitus, overweight or obese patients had a lower risk of major adverse CV events (MACE) and all-cause mortality than those with normal-weight patients [11]. However, there is limited evidence regarding the long-term prognosis in patients with established CV disease. Therefore, we investigated the association of obesity and diabetes with CV outcomes in patients treated with acute coronary syndrome (ACS).

## Methods

### Study population

This study used data from the nationwide administrative claims-based databases of the Korean National Health Insurance Service (NHIS), which covers > 98% of the entire Korean population. The NHIS-Health Screening Cohort is a dataset with a random sample of 10% of the population aged 40–79 years who completed a National Health Screening test in 2002 or 2003. The database provides information regarding the demographic characteristics, medical claims (including diagnostic and treatment codes), health surveys, physical examinations, and biochemical tests of 514,866 participants. The diagnostic codes are based on the International Classification of Diseases, 10th revision (ICD-10) [12].

**Fig. 1:**
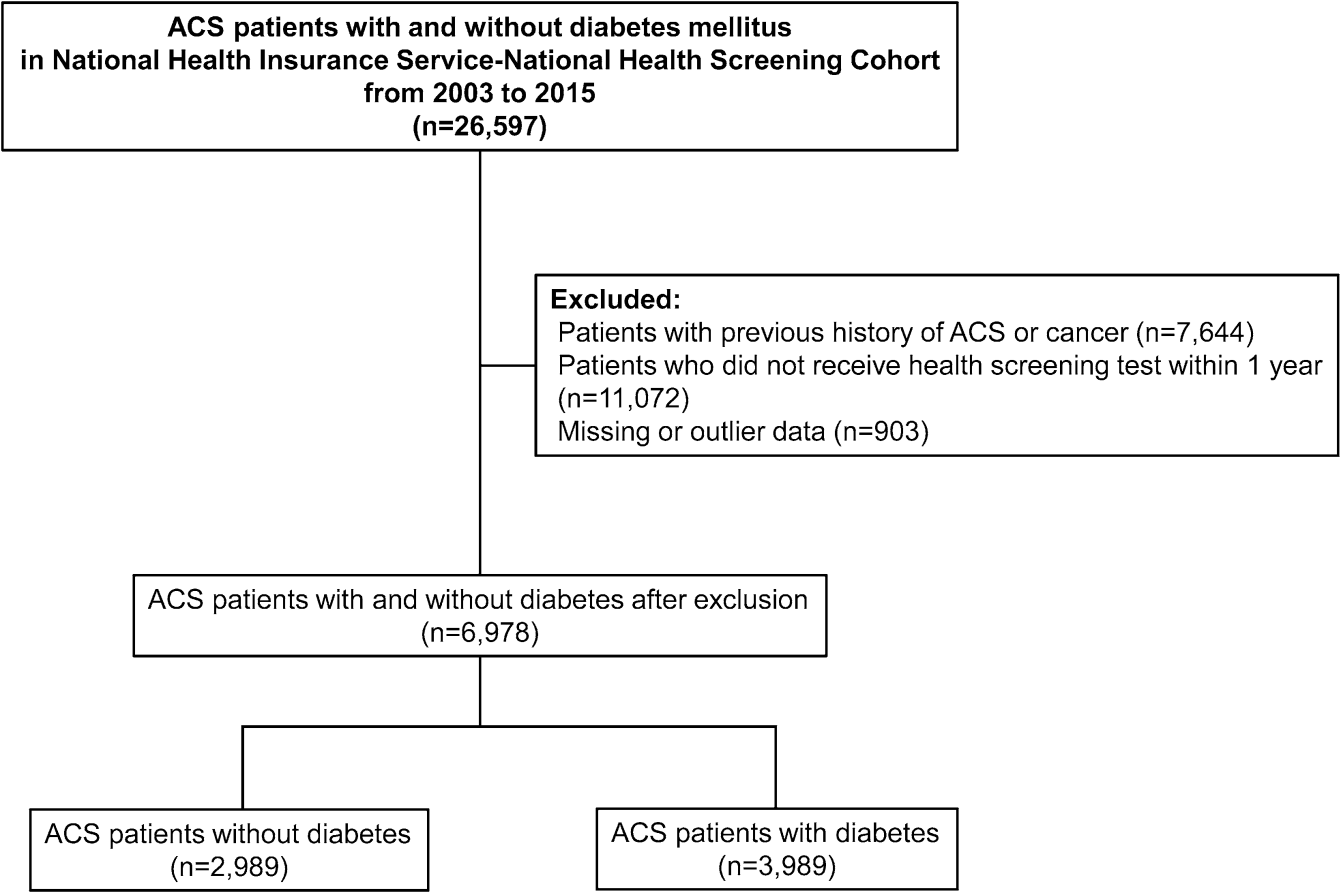
Flowchart of the study population. ACS, acute coronary syndrome

This study included patients who were first hospitalized with ACS (ICD-10 codes: I20–22) between 2002 and 2015. Patients with a previous history of cancer or outpatient diagnosis with ACS before the first hospitalization were excluded. Patients whose data were unavailable for analysis or not received the health screening test within 1 year before the first hospitalization for ACS were also excluded (Fig. 1). The follow-up period was defined as the time from the index date (date of hospital discharge) to the each outcome event, date of death, or end of the study period (December 31, 2015), whichever came first. The study protocol was reviewed and approved by the Institutional Review Board of Ajou University Hospital (AJIRB-MED-EXP-17-253). The requirement for informed consent was waived as the data in this database were de-identified.

### Outcomes and covariates

The following demographic and anthropometric data were collected 1 year before the first hospitalization date for ACS: age, sex, height, weight, and blood pressure. BMI was defined as weight (kg) divided by height in meter squared (m^2^) and categorized according to the Asian-specific criteria as [13]: underweight (< 18.5 kg/m^2^), normal weight (18.5–22.9 kg/m^2^), overweight (23.0–24.9 kg/m^2^), obese class I (25.0–29.9 kg/m^2^), and obese class II (≥ 30.0 kg/m^2^). Total cholesterol and glucose levels were collected from baseline fasting blood sample analysis. Smoking status (smoker, former smoker, or nonsmoker), alcohol consumption (low, middle or high), and physical activity (low, middle, or high) were obtained from self-reported questionnaires. Household income was categorized into three groups (lowest 30%, middle 40%, and highest 30%). The prescribed drugs were categorized using the Anatomical Therapeutic Chemical (ATC) codes: antihypertensive agents (C07–C09), statins (C10AA), and antiplatelet agents (B01A). Baseline comorbidities included a previous history of stroke (ICD-10 code I50) and heart failure (ICD-10 codes I60–I64). Diabetes mellitus was defined using the following criteria: diagnosed with ICD-10 codes (E10–E14) more than twice, receiving glucose-lowering agents (ATC code A10B) for > 30 days, receiving insulin (ATC code A10A) as an outpatient, or fasting blood glucose level of ≥ 126 mg/dL.

The primary outcome was MACE—CV death (ICD-10 codes I00–I99), MI (ICD-10 codes I21–I22), and stroke (ICD-10 codes I60–I64). The secondary outcomes were individual components of MACE, hospitalization for heart failure (HHF; ICD-10 code I50), and all-cause death. All-cause and CV death were defined by death status in the NHIS database, which was linked to the National Death Registry using unique resident registration numbers.

### Statistical analysis

All categorical variables are presented as frequencies and percentages. Normally distributed data were presented as mean ± standard deviation, whereas nonparametric data are presented as median and interquartile range by BMI. Differences between patients with and without diabetes were analyzed using the Student’s *t* test for continuous variables and the Chi square test for categorical variables. Cox proportional hazard regression analyses were performed to identify the association of BMI with the primary and secondary outcomes according to the presence of diabetes, calculating hazard ratio (HR) and 95% confidence interval (CI) and adjusting for the following potential confounders: sex, age, systolic blood pressure, fasting glucose level, total cholesterol level, alcohol consumption, smoking status, physical activity, household income, use of antihypertensive agents, use of statins, use of antiplatelet agents, previous history of heart failure, previous history of stroke, and index year. All analyses were conducted using SAS version 9.4 (SAS Institute Inc., Cary, NC, USA).

## Results

The study initially enrolled 26,597 patients hospitalzed with ACS, and 6978 participants fulfilled the inclusion criteria (Fig. 1). The baseline characteristics according to diabetes status are summarized in Table 1. Compared with patients without diabetes, those with diabetes (n = 3989, 57.1%) were older, were more likely to be females, and had higher BMI and systolic blood pressures. Patients with diabetes were less likely to be current smokers and had low alcohol consumption compared with those without diabetes; however, they had more frequent comorbidities and more frequently used concomitant medications. The baseline characteristics of patients with and without diabetes stratified by BMI are summarized (Additional file 1: Table S1). Obese patients were more likely to be younger, males, and physically active, and current smokers and more frequently used medications than normal-weighted patients.

**Table 1:** Baseline characteristics by diabetes status

	Without diabetes (n = 2989)	With diabetes (n = 3989)	*P* value
Age, years	60.8 ± 9.7	64.4 ± 9.5	< 0.001
Women, n (%)	1038 (34.7)	1528 (38.3)	0.002
Body mass index, kg/m^2^	24.2 ± 2.9	24.6 ± 3.1	< 0.001
Systolic blood pressure, mmHg	130.1 ± 17.7	131.7 ± 17.9	< 0.001
Diastolic blood pressure, mmHg	80.4 ± 11.3	80.0 ± 11.0	0.073
Total cholesterol, mg/dL	203.7 ± 40.1	201.5 ± 44.6	0.030
Fasting glucose, mg/dL	94.0 (86.0–102.0)	106.0 (92.0–131.0)	< 0.001
Clinical diagnosis, n (%)			
Myocardial infarction	1565 (52.4)	1946 (48.8)	0.003
Unstable angina	1424 (47.6)	2043 (51.2)	
Smoking status, n (%)			
Never	1745 (58.4)	2463 (61.7)	< 0.001
Former	429 (14.3)	630 (15.8)	
Current	815 (27.3)	896 (22.5)	
Alcohol consumption, n (%)			
Low	2032 (68.0)	2996 (75.1)	< 0.001
Middle	874 (29.2)	904 (22.7)	
High	83 (2.8)	89 (2.2)	
Physical activity, n (%)			
Low	1007 (33.7)	1066 (26.7)	< 0.001
Middle	1567 (52.4)	2366 (59.3)	
High	415 (13.9)	557 (14.0)	
Household income, n (%)			
Lower 30%	611 (20.4)	921 (23.1)	0.030
Mid 40%	996 (33.3)	1283 (32.2)	
Upper 30%	1382 (46.2)	1785 (44.7)	
Comorbidities, n (%)			
Heart failure	376 (12.6)	830 (20.8)	< 0.001
Stroke	445 (14.9)	967 (24.2)	< 0.001
Concurrent medication, n (%)			
ACEi or ARB	2166 (72.5)	3377 (84.7)	< 0.001
Beta-blockers	2268 (75.9)	3383 (84.8)	< 0.001
Calcium channel blocker	2120 (70.9)	3229 (80.9)	< 0.001
Statin	2208 (73.9)	3382 (84.8)	< 0.001
Antiplatelet agents	2056 (68.8)	3204 (80.3)	< 0.001

ACEi, angiotensin-converting enzyme inhibitor; ARB, angiotensin receptor blocker

### Association of diabetes with CV outcomes

During a mean follow-up of 5.4 ± 3.7 years (median, 4.9 years), 1633 (23.4%) MACE and 1023 (14.7%) deaths occurred. ACS patients with diabetes had a higher risk of MACE (event rates 5.81 vs. 4.01 per 100 person-years, HR 1.49, 95% CI 1.34–1.65) and its individual components than those without diabetes (CV death: HR 1.25, 95% CI 1.03–1.53; MI: HR 1.26, 95% CI 1.09–1.50; stroke: HR 2.06, 95% CI 1.75–2.42). These associations were attenuated after adjustment for confounding variables, and remained significant for MACE and stroke (MACE: HR 1.22, 95% CI 1.09–1.37; stroke: HR 1.50, 95% CI 1.26–1.79). Patients with diabetes also had higher event rates and risks of HHF and all-cause death than patients without diabetes (HHF: event rates 2.40 vs. 1.15, HR 2.13, 95% CI 1.79–2.55; all-cause death: event rates 3.05 vs. 2.25, HR 1.40, 95% CI 1.23–1.60). This association remained significant after adjustment for confounding variables (HHF: HR 1.47, 95% CI 1.22–1.78; all-cause death: HR 1.28, 95% CI 1.11–1.49) (Table 2).

**Table 2:** Hazard ratio of cardiovascular outcomes in patients with acute coronary syndrome by diabetes status

	Without diabetes (n = 2989)			With diabetes (n = 3989)			Unadjusted HR (95% CI)	Adjusted HR (95% CI)^a^
	Person-years	No. of events	Event rate (per 100 PY)	Person-years	No. of events	Event rate (per 100 PY)		
MACE	13,057	524	4.01	19,096	1109	5.81	1.49 (1.34–1.65)	1.22 (1.09–1.37)
Cardiovascular death	14,471	153	1.06	22,913	286	1.25	1.25 (1.03–1.53)	1.15 (0.92–1.43)
Myocardial infarction	13,542	272	2.01	20,734	500	2.41	1.26 (1.09–1.50)	1.05 (0.89–1.23)
Stroke	13,863	196	1.41	20,812	592	2.84	2.06 (1.75–2.42)	1.50 (1.26–1.79)
Hospitalization for heart failure	14,103	162	1.15	21,385	513	2.40	2.13 (1.79–2.55)	1.47 (1.22–1.78)
All-cause death	14,471	325	2.25	22,913	698	3.05	1.40 (1.23–1.60)	1.28 (1.11–1.49)

*CI* confidence interval, *HR* hazard ratio, *MACE* major adverse cardiovascular events, *PY* person-years

^a^Adjusted for sex, age, body mass index, systolic blood pressure, fasting glucose, total cholesterol, alcohol consumption, smoking status, physical activity, household income, concurrent medications, comorbidities, and index year

### Association of BMI and diabetes with CV outcomes

**Fig. 2:**
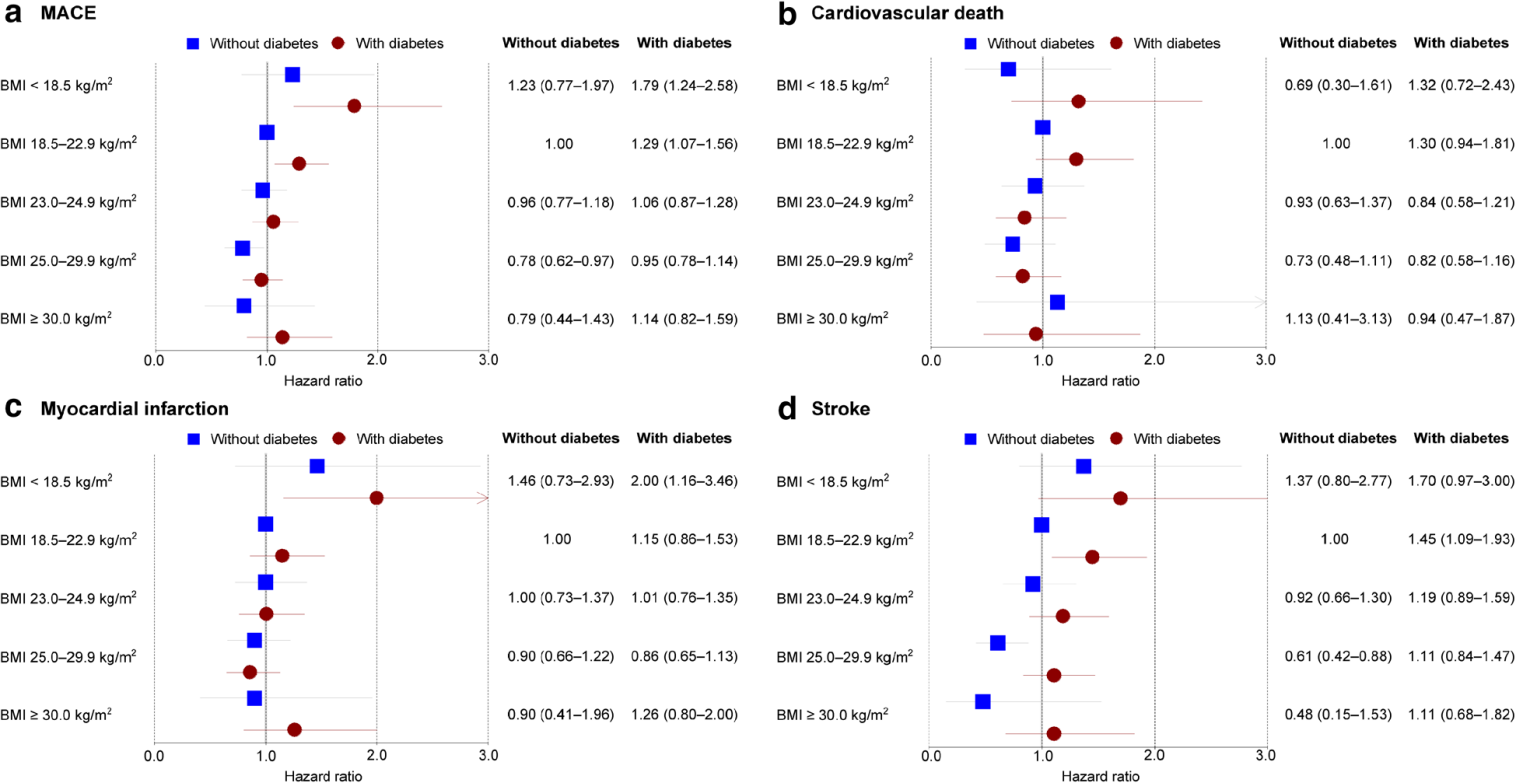
Hazard ratio of cardiovascular outcomes in patients with acute coronary syndrome by diabetes status and body mass index. Adjusted for sex, age, body mass index, systolic blood pressure, fasting glucose, total cholesterol, alcohol consumption, smoking status, physical activity, household income, concurrent medications, comorbidities, and index year.* BMI* body mass index, * MACE* major adverse cardiovascular events

The risks of the CV outcomes by BMI and diabetes status in ACS patients are described in Fig. 2. After adjustment for confounding variables, compared to normal-weight patients without diabetes (reference group), obese class I patients with and without diabetes had a lower risk of MACE, but only significant in patients without diabetes (with diabetes: HR 0.95, 95% CI 0.78–1.14; without diabetes: HR 0.78, 95% CI 0.62–0.97). Regarding individual components of MACE except for stroke, obese class I patients with and without diabetes tend to be a lower risk with no statistical significance. In terms of stroke, obese class I patient without diabetes was associated with a lower risk, on the other hand, those with diabetes were not (with diabetes: HR 1.11, 95% CI 0.84–1.47; without diabetes: HR 0.61, 95% CI 0.42–0.88). Among the secondary outcomes with HHF and all-cause death, obese patients showed similar results to the reference group, but obese class I patients without diabetes had a lower risk of HHF (HR 0.62, 95% CI 0.42–0.92) (Additional file 1: Fig. S1).

In contrast, underweight patients with and without diabetes had a higher risk of MACE compared to the reference group, but only significant in patients with diabetes (with diabetes: HR 1.79, 95% CI 1.24–2.58; without diabetes: HR 1.23, 95% CI 0.77–1.97). In patients with diabetes, the underweight BMI was associated with a higher risk of individual components of MACE, but only significant for the risk of MI (HR 2.00. 95% CI 1.16–3.46). In patients without diabetes, underweight tend to increase the risk of those events except for CV death, with no statistical significance. In terms of all-cause death among secondary outcomes, underweight patients with and without diabetes had a significantly higher risk (with diabetes: HR 2.07, 95% CI 1.43–3.00; without diabetes: HR 2.07, 95% CI 1.40–3.07).

The subgroup analyses stratified by sex, age, smoking status, and clinical diagnosis were shown in Additional file 3: Fig. S2. In all subgroups, obese I class patients without diabetes had a lower risk of MACE, but only significant in female, elderly (≥ 65 years), and hospitalization for unstable angina patients. In contrast, underweight patients with diabetes had a higher risk of MACE, but only significant in male, younger (< 65 years), current smoker, and hospitalization for MI patients.

## Discussion

This study showed that obesity and diabetes affected the long-term outcomes of patients with ACS. While diabetes deteriorated clinical outcomes, obesity ameliorated the results; the “obesity paradox” was obvious in ACS patients, especially in those without diabetes. Obesity decreased the risk of MACE driven primarily by stroke and reduced the rate of HHF exclusively in patients without diabetes. These were more evident in female, elderly and hospitalized for unstable angina patients without diabetes. All-cause and CV mortality seemed to be lowered in overweight and obese patients with and without diabetes. In contrast, underweight increased the risk of all-cause death in patients with and without diabetes, and also increased the risk of MACE in patients with diabetes.

### Obesity paradox

Obesity increases the risk of CV disease via dysregulated metabolism sharing the common mechanism with diabetes (e.g., insulin resistance and inflammation) [14]. In a study with 390 patients underwent surgical endarterectomy, obesity (BMI ≥ 30 kg/m^2^) was associated with the vulnerable carotid plaque, particularly in males aged < 70 years [15]. The SPUM-ACS (Special Program University Medicine-Acute Coronary Syndrome) study showed that obese patients with ACS (BMI ≥ 30 kg/m^2^) had a higher risk of MACE than normal-weight patients, which was driven by the increased risk of repeat revascularization [6].

However, in contrast to the biological effect of obesity, overweight or obese patients show a better prognosis than normal-weight patients, which is called the “obesity paradox”. A variety of mechanism has been proposed to explain this phenomenon. BMI has been debated for a reliable obesity parameter without the discrimination between lean body mass and fat mass [3, 14, 16]. Body fat distribution may be more important than overall adiposity as visceral fat is a strong predictor of metabolic derangement [3, 14]. Obesity is related to the increased muscle mass and strength represented as physical activity or cardiorespiratory fitness [3]. The paradoxical effect of obesity might be attributable to unmeasured confounding factors or biases [3, 16].

In a meta-analysis study, obese patients with coronary artery disease had no increased risk for all-cause and CV death, which was most evident in BMI 25.0–29.9 kg/m^2^ [17]. In particular, the obesity paradox has been identified in elderly patients. The higher BMI was associated with better prognosis and preserved gait speed in Japanese elderly patients with CV disease [18]. In the study with 2120 Japanese MI patients treated with primary coronary intervention, obesity was associated with the lower mortality only in elderly patients [19]. Given the increased catabolism in CV disease, obesity could play a role as the metabolic reserve [3, 20]. The finding of coronary angiography showed better characteristics in obese patients with ST-elevation MI [21]. The current study also showed obesity paradox in the overall population; overweight or obese patients had a favorable outcome of MACE compared with the normal-weight group (Additional file 1: Table S2). Not only CV and all-cause mortality, the risk of stroke and HHF were also lowered in obese patients than in those with normal weight. A previous study of 5202 patients with a previous history of CV disease reported a consistent finding that obesity was protective for clinical outcomes including stroke and HHF. The risks of stroke and HHF increased by 10% and 5%, respectively, with the decreased weight [22].

### Obesity paradox in diabetes

Patients with diabetes not only have a higher risk for developing coronary heart disease, but their prognosis worsens after ACS. The SPUM-ACS cohort revealed that both the entry hyperglycemia and diabetes affected the short- and long-term MACE [6]. In a previous cohort study, patients with diabetes had a lower socioeconomic status than those without diabetes, which would also affect the adverse CV results [9]. We also found that in patients with ACS, diabetes was associated with an increased risk of CV outcomes. However, it is unclear whether the presence of diabetes affects the relationship between obesity and CV outcomes.

The PROactive (PROspective pioglitAzone Clinical Trial In macroVascular Events) study revealed that patients with diabetes and CV comorbidity had the risk of all-cause and CV death, which increased by 13% and 7% for each 1% of weight loss [22]. However, the increasing BMI enhanced the clinical outcomes during a long follow-up period in heart failure patients without diabetes, which was not observed in those with diabetes [23, 24]. In the MONICA/KORA (MONItoring of Trends and Determinants in CArdiovascular Diseases’ Augsburg/Cooperative Health Research in the Region of Augsburg) from German population-based acute MI registry, the protective effect of overweight and obesity on all-cause mortality was shown only in patients without diabetes, but not in those with diabetes [25]. The findings of the present study also did not support obesity paradox in patients with diabetes. Obese patients with diabetes, compared with normal-weight patients without diabetes, neither showed a lower risk of CV outcomes.

The cardiometabolic consequences of obesity may have a more relevant impact on CV outcomes than obesity. A study on the population with CV disease demonstrated that cardiometabolic dysfunction increased the risk of CV morbidity and all-cause mortality. Even in comparison with normal-weight patients with cardiometabolic dysfunction, overweight and obese patients had a comparable prognosis of CV disease [26]. A propensity-matched study of 7788 patients with heart failure also reported a consistent result: the paramount difference in mortality between obese patients with and without diabetes [24]. Diabetes seems to be a stronger predictor of CV outcomes than obesity, which can negate the positive impact of obesity on the outcomes. Thus, the impaired cardiometabolic function, such as diabetes, might offset the protective role of obesity.

### Clinical implication

The present study adds to the accumulating evidence that “obesity paradox” is obvious in patients with CV disease. It is focused on the relationship between diabetes and BMI, that diabetes deteriorates the prognosis of ACS patients for the long-term duration in the Korean population while obesity was protective. Our findings showed that the aim of treatment in ACS patients with and without diabetes should be to prevent or control cardiometabolic complications and not merely weight reduction. In cardiometabolic dysfunction, the weight control without the management for CV risk factors did not have survival benefit [5]. Meanwhile, lowering the weight should not be underemphasized as intentional weight loss is still associated with an improvement of comorbidities and long-term prognosis in CV disease [20].

## Limitations

This study has some limitations. First, the patients enrolled in the study were limited to those aged 40-79 years, as the NHIS-Screening Cohort basically did not supply the data of those aged < 40 year and those ≥ 80 years due to the very low proportion in the cohort and the very low response rate, respectively [12]. Thus, we did not consider the impact of age on obesity paradox in ACS patients, specifically in elderly patients. Second, these results were based on a single assessment of BMI, which limits our ability to address incorporate temporal changes. In addition, we did not investigate other obesity-related indices such as cardiorespiratory fitness and central obesity. In comparison to BMI, physical activity or cardiorespiratory fitness were more potent predictors for CV disease [3, 16]. A meta-analysis with 102,980 participants showed that each 1 metabolic equivalent increase in cardiorespiratory fitness decreased all-cause and CV death by 13% and 15%, respectively [27]. Central obesity (e.g., waist-to-hip ratio) was also more related to the clinical outcomes [25]. Also, information relevant to diabetes, such as duration, type, severity, or treatment, was not examined. Thus, the possibility of residual confounding effects remains. Third, the data obtained from the claims data could not exclude the possibility of a diagnosis code inaccuracy and disease misclassification. Recent Korean studies that compared diagnosis from claim databases with medical records observed overall accuracy rates of 92.0% for MI, and 90.5% for ischemic stroke [28]. There was a possibility of non-differential misclassification which bias the risk ratio toward the null.

## Conclusion

In patients diagnosed with ACS, obesity had a protective effect on MACE, especially in patients without diabetes. The clinical practice might focus on improving the cardiometabolic profile rather than just losing the body weight for a better long-term prognosis.

## Supplementary information

**Additional file 1: Table** **S1.** Baseline characteristics of patients with and without diabetes stratified by body mass index. **Table S2**. Clinical outcomes of patients stratified by body mass index.

**Additional file 2: Figure S1.** Hazard ratio of (**a**) hospitalization for heart failure and (**b**) all-cause death in patients with acute coronary syndrome according to body mass index and diabetes status. Adjusted for sex, age, body mass index, systolic blood pressure, fasting glucose, total cholesterol, alcohol consumption, smoking status, physical activity, household income, concurrent medications, comorbidities, and index year.

**Additional file 3: Figure S2.** Subgroup analysis for hazard ratio of major adverse cardiovascular events in patients with acute coronary syndrome according to body mass index and diabetes status. Subgroup was stratified by (**a**, **b**) sex, (**c**, **d**) age, (**e**, **f**) smoking status, and (**g**, **h**) clinical diagnosis. Adjusted for sex, age, body mass index, systolic blood pressure, fasting glucose, total cholesterol, alcohol consumption, smoking status, physical activity, household income, concurrent medications, comorbidities, and index year.

## Data Availability

Data cannot be shared publicly as the access of National Health Insurance Service (NHIS) data is available only at the NHIS center, Wonju, Republic of Korea. The contact information for the NHIS center of Republic of Korea is as follows: +82-33-736-2431-3 (Tel) and https://nhiss.nhis.or.kr (website).

## Abbreviations

ACS: Acute coronary syndrome
ATC: Anatomical Therapeutic Chemical
BMI: Body mass index
CI: Confidence interval
CV: Cardiovascular
HHF: Hospitalization for heart failure
HR: Hazard ratio
ICD: International Classification of Diseases
MACE: Major adverse cardiovascular event
MI: Myocardial infarction
NHIS: National Health Insurance Service
