# Supplementary material for: Body mass index and cardiovascular outcomes in patients with acute coronary syndrome by diabetes status: the obesity paradox in a Korean national cohort study
Source: Cardiovasc Diabetol. 2020 Nov 10;19:191. doi: 10.1186/s12933-020-01170-w (PMC7656714; doi:10.1186/s12933-020-01170-w)
Supplement: Supplementary file 1 — Additional file 1: Table S1. Baseline characteristics of patients with and without diabetes stratified by body mass index. Table S2. Clinical outcomes of patients stratified by body mass index. [file 12933_2020_1170_MOESM1_ESM.pdf]

Supplementary table 1. Baseline characteristics of patients with and without diabetes stratified by body mass index.

	Without diabetes					With diabetes				
	<18.5 (n=68)	18.5–22.9 (n=889)	23.0–24.9 (n=886)	25.0–29.9 (n=1065)	≥30.0 (n=81)	<18.5 (n=85)	18.5–22.9 (n=1067)	23.0–24.9 (n=1096)	25.0–29.9 (n=1572)	≥30.0 (n=169)
Age, years	70.2 ± 10.6	61.8 ± 10.4	60.7 ± 9.2	59.4 ± 9.0	61.1 ± 8.8	70.6 ± 10.9	65.9 ± 9.5	64.1 ± 9.2	63.4 ± 9.3	63.8 ± 9.2
Female, n (%)	20 (29.4)	292 (32.8)	319 (36.0)	364 (34.2)	43 (53.1)	35 (41.2)	414 (38.8)	375 (34.2)	611 (38.9)	93 (55.0)
Body mass index, kg/m^2	17.4 ± 0.9	21.4 ± 1.1	24.0 ± 0.6	26.7 ± 1.3	31.5 ± 1.8	17.4 ± 1.0	21.4 ± 1.1	24.0 ± 0.6	26.8 ± 1.3	31.9 ± 1.9
Systolic blood pressure, mmHg	131.5 ± 22.0	128.3 ± 17.5	129.6 ± 18.3	131.7 ± 17.2	133.5 ± 14.8	125.0 ± 19.5	130.3 ± 18.5	131.1 ± 17.5	133.0 ± 17.2	135.7 ± 19.8
Diastolic blood pressure, mmHg	79.6 ± 12.8	79.1 ± 11.3	80.3 ± 11.5	81.5 ± 11.1	83.5 ± 8.4	76.7 ± 12.1	78.8 ± 11.4	79.4 ± 10.6	81.1 ± 10.8	82.2 ± 11.6
Total cholesterol, mg/dL	189.2 ± 47.4	198.9 ± 39.0	203.0 ± 38.0	208.7 ± 40.8	210.9 ± 47.7	181.8 ± 45.9	197.8 ± 44.3	201.1 ± 43.7	205.4 ± 44.6	201.1 ± 46.0
Fasting glucose, mg/dL	91.5 (79.0–104.0)	92.0 (85.0–100.0)	93.0 (85.0–100.0)	95.0 (87.0–103.0)	95.0 (87.0–104.0)	100.0 (89.0–121.0)	105.0 (92.0–134.0)	105.0 (91.0–130.0)	107.0 (93.0–129.0)	108.0 (94.0–135.0)
Clinical diagnosis, n (%)										
Myocardial infarction	43 (63.2)	518 (58.3)	442 (49.9)	520 (48.8)	42 (51.9)	60 (70.6)	548 (51.4)	530 (48.4)	727 (46.3)	81 (47.9)
Unstable angina	25 (36.8)	371 (41.7)	444 (50.1)	545 (51.2)	39 (48.1)	25 (29.4)	519 (48.6)	566 (51.4)	845 (53.7)	88 (52.1)
Smoking status, n (%)										
Never	37 (54.4)	510 (57.4)	505 (57.0)	631 (59.2)	62 (76.5)	44 (51.8)	654 (61.3)	657 (59.9)	987 (62.8)	121 (71.6)
Former	3 (4.4)	121 (13.6)	126 (14.2)	169 (15.9)	10 (12.4)	12 (14.1)	154 (14.4)	175 (16.0)	266 (16.9)	23 (13.6)
Current	28 (41.2)	258 (29.0)	255 (28.8)	265 (24.9)	9 (11.1)	29 (34.1)	259 (24.3)	264 (24.1)	319 (20.3)	25 (14.8)
Alcohol consumption, n (%)										
Low	47 (69.1)	597 (67.1)	587 (66.3)	733 (68.8)	68 (84.0)	69 (81.2)	806 (75.5)	805 (73.4)	1183 (75.2)	133 (78.7)
Middle	18 (26.5)	261 (29.4)	272 (30.7)	310 (29.1)	13 (16.0)	12 (14.1)	234 (21.9)	267 (24.4)	355 (22.6)	36 (21.3)
High	3 (4.4)	31 (3.5)	27 (3.0)	22 (2.1)	0 (0.0)	4 (4.7)	27 (2.5)	24 (2.2)	34 (2.2)	0 (0.0)
Physical activity, n (%)										
Low	34 (50.0)	307 (34.5)	299 (33.7)	342 (32.1)	25 (30.9)	25 (29.4)	299 (28.0)	270 (24.6)	428 (27.2)	44 (26.0)

Middle	25 (36.8)	467 (52.5)	457 (51.6)	569 (53.4)	49 (60.5)	53 (62.4)	614 (57.5)	671 (61.2)	924 (58.8)	104 (61.5)
High	9 (13.2)	115 (12.9)	130 (14.7)	154 (14.5)	7 (8.6)	7 (8.2)	154 (14.4)	155 (14.1)	220 (14.0)	21 (12.4)
Household income, n (%)										
Lower 30%	29 (42.7)	194 (21.8)	165 (18.6)	204 (19.2)	19 (23.5)	25 (29.4)	238 (22.3)	276 (25.2)	338 (21.5)	44 (26.0)
Mid 40%	19 (27.9)	318 (35.8)	310 (35.0)	322 (30.2)	27 (33.3)	28 (32.9)	355 (33.3)	357 (32.6)	482 (30.7)	61 (36.1)
Upper 30%	20 (29.4)	377 (42.4)	411 (46.4)	539 (50.6)	35 (43.2)	32 (37.7)	474 (44.4)	463 (42.2)	752 (47.8)	64 (37.9)
Concurrent medication, n (%)										
ACEi or ARB	43 (63.2)	570 (64.1)	656 (74.0)	830 (77.9)	67 (82.7)	66 (77.6)	867 (81.3)	912 (83.2)	1380 (87.8)	152 (89.9)
Beta blockers	42 (61.8)	641 (72.1)	657 (74.2)	859 (80.7)	69 (85.2)	60 (70.6)	886 (83.0)	918 (83.8)	1368 (87.0)	151 (89.3)
Calcium channel blockers	45 (66.2)	584 (65.7)	623 (70.3)	804 (75.5)	64 (79.0)	60 (70.6)	802 (75.2)	876 (79.9)	1326 (84.4)	147 (87.0)
Statin	35 (51.5)	570 (64.1)	694 (78.3)	844 (79.2)	65 (80.2)	47 (55.3)	857 (80.3)	949 (86.6)	1383 (88.0)	146 (86.4)
Antiplatelet agents	37 (54.4)	561 (63.1)	609 (68.7)	784 (73.6)	65 (80.2)	59 (69.4)	827 (77.5)	896 (81.8)	1277 (81.2)	145 (85.8)

ACEi, angiotensin converting enzyme inhibitor; ARB, angiotensin receptor blocker.

Supplementary table 2. Hazard ratio of cardiovascular outcomes in patients with acute coronary syndrome by body mass index

	No. of person	Person-years	No. of events	Event rate (per 100 PY)	Unadjusted HR (95% CI)	Adjusted* HR (95% CI)
MACE						
< 18.5	153	473	56	11.84	1.73 (1.31-2.28)	1.33 (1.00-1.76)
18.5–22.9	1956	8489	519	6.11	1.00	1.00
23.0–24.9	1982	9220	462	5.01	0.84 (0.74-0.95)	0.86 (0.76-0.98)
25.0–29.9	2637	12858	538	4.18	0.71 (0.63-0.80)	0.75 (0.66-0.85)
≥ 30.0	250	1113	58	5.21	0.86 (0.65-1.12)	0.87 (0.66-1.14)
Cardiovascular death						
< 18.5	153	580	19	3.28	1.72 (1.07-2.76)	0.88 (0.54-1.43)
18.5–22.9	1956	9924	170	1.71	1.00	1.00
23.0–24.9	1982	10851	112	1.03	0.62 (0.49-0.78)	0.74 (0.58-0.94)
25.0–29.9	2637	14724	124	0.84	0.51 (0.40-0.64)	0.66 (0.52-0.84)
≥ 30.0	250	1307	14	1.07	0.63 (0.37-1.09)	0.82 (0.47-1.43)
Myocardial infarction						
< 18.5	153	516	25	4.85	1.78 (1.18-2.69)	1.62 (1.07-2.47)
18.5–22.9	1956	9065	222	2.45	1.00	1.00
23.0–24.9	1982	9918	225	2.27	0.95 (0.79-1.14)	0.92 (0.77-1.11)
25.0–29.9	2637	13585	268	1.97	0.83 (0.70-0.99)	0.80 (0.66-0.95)
≥ 30.0	250	1192	32	2.68	1.10 (0.76-1.59)	1.06 (0.72-1.54)
Stroke						
< 18.5	153	520	24	4.62	1.56 (1.03-2.38)	1.24 (0.81-1.90)
18.5–22.9	1956	9185	254	2.77	1.00	1.00
23.0–24.9	1982	9978	227	2.27	0.83 (0.70-1.00)	0.84 (0.70-1.01)

25.0–29.9	2637	13787	258	1.87	0.69 (0.58-0.82)	0.73 (0.61-0.87)
≥ 30.0	250	1204	25	2.08	0.75 (0.50-1.13)	0.72 (0.47-1.09)
Hospitalization for heart failure						
< 18.5	153	560	18	3.21	1.30 (0.81-2.11)	1.03 (0.63-1.67)
18.5–22.9	1956	9340	223	2.39	1.00	1.00
23.0–24.9	1982	10393	182	1.75	0.74 (0.61-0.90)	0.76 (0.62-0.93)
25.0–29.9	2637	59409	222	0.37	0.67 (0.56-0.81)	0.69 (0.57-0.84)
≥ 30.0	250	1235	30	2.43	1.02 (0.70-1.49)	0.84 (0.57-1.24)
All-cause death						
< 18.5	153	580	72	12.42	3.08 (2.39-3.96)	1.64 (1.26-2.13)
18.5–22.9	1956	9924	377	3.80	1.00	1.00
23.0–24.9	1982	10851	248	2.29	0.61 (0.52-0.71)	0.73 (0.62-0.86)
25.0–29.9	2637	14724	300	2.04	0.55 (0.47-0.63)	0.73 (0.63-0.86)
≥ 30.0	250	1307	26	1.99	0.53 (0.35-0.78)	0.75 (0.51-1.13)

*Adjusted for sex, age, body mass index, systolic blood pressure, fasting glucose, total cholesterol, alcohol consumption, smoking status, physical activity, household income, concurrent medications, comorbidities, and index year.

CI, confidence interval;HR, hazard ratio; MACE, major adverse cardiovascular events; PY, person-years
