# Supplementary figures and images for: Body mass index and cardiovascular outcomes in patients with acute coronary syndrome by diabetes status: the obesity paradox in a Korean national cohort study
Source: Cardiovasc Diabetol. 2020 Nov 10;19:191. doi: 10.1186/s12933-020-01170-w (PMC7656714; doi:10.1186/s12933-020-01170-w)

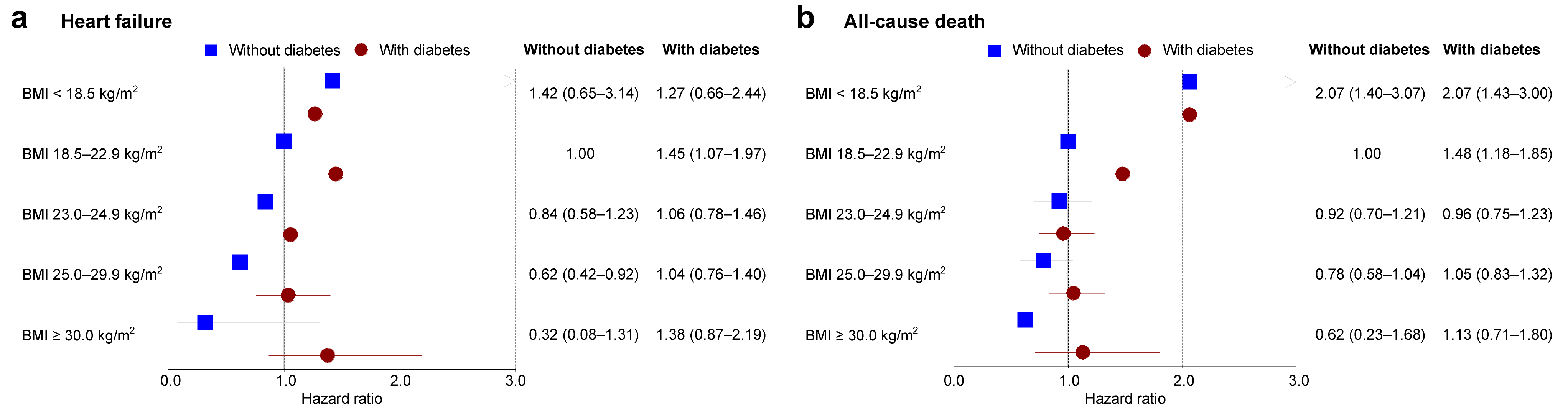

Supplement: Supplementary file 2 — Additional file 2: Figure S1. Hazard ratio of (a) hospitalization for heart failure and (b) all-cause death in patients with acute coronary syndrome according to body mass index and diabetes status. Adjusted for sex, age, body mass index, systolic blood pressure, fasting glucose, total cholesterol, alcohol consumption, smoking status, physical activity, household income, concurrent medications, comorbidities, and index year. [file 12933_2020_1170_MOESM2_ESM.tif]

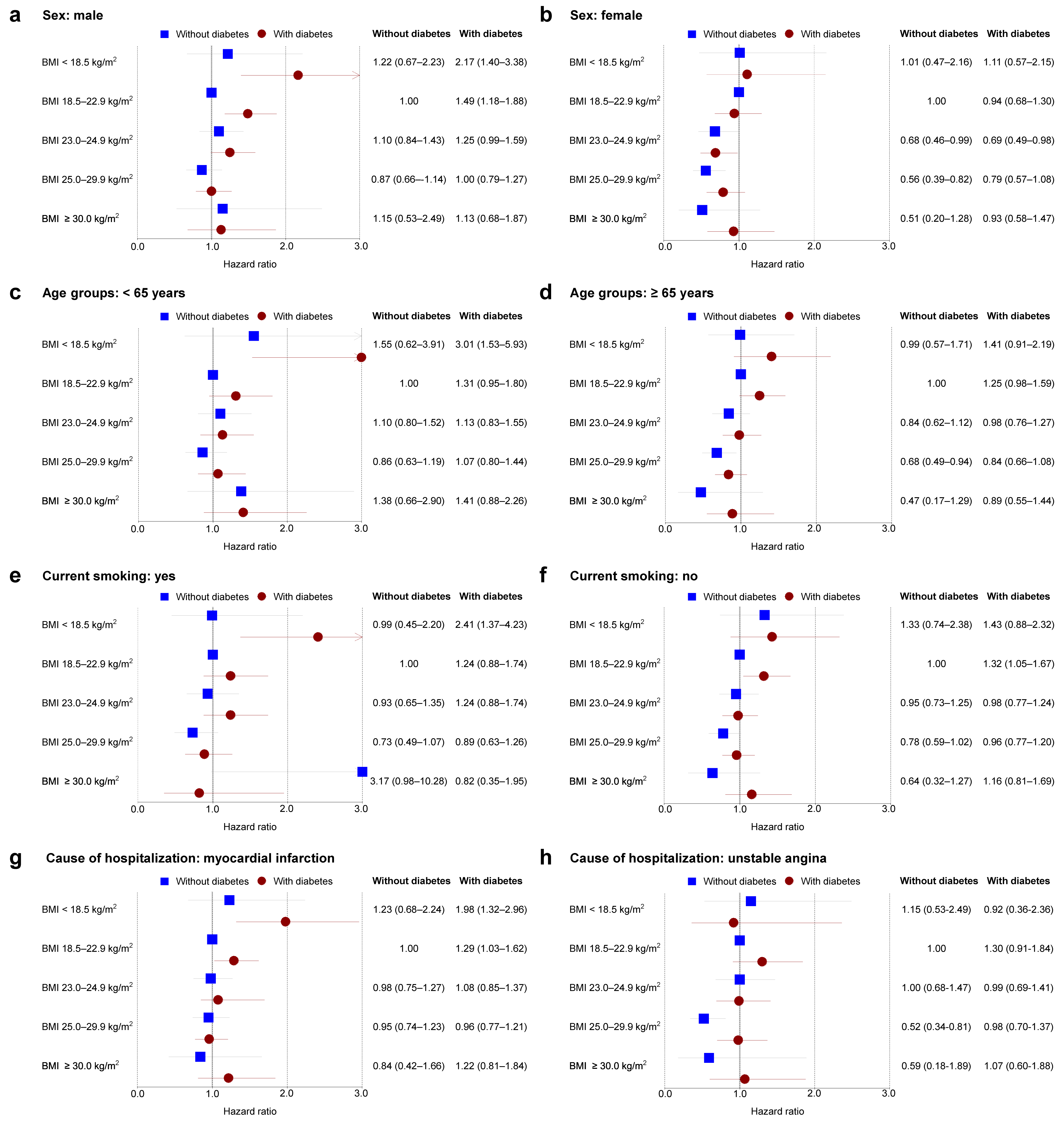

Supplement: Supplementary file 3 — Additional file 3: Figure S2. Subgroup analysis for hazard ratio of major adverse cardiovascular events in patients with acute coronary syndrome according to body mass index and diabetes status. Subgroup was stratified by (a, b) sex, (c, d) age, (e, f) smoking status, and (g, h) clinical diagnosis. Adjusted for sex, age, body mass index, systolic blood pressure, fasting glucose, total cholesterol, alcohol consumption, smoking status, physical activity, household income, concurrent medications, comorbidities, and index year. [file 12933_2020_1170_MOESM3_ESM.tif]
